# Evaluation of trait preferences and effect of parity, season and lactation stage on production performance of indigenous dairy cow in kaffa zone, southwest Ethiopia

**DOI:** 10.1016/j.heliyon.2023.e22380

**Published:** 2023-11-17

**Authors:** Regasa Begna, Yakob Asfaw, Worku Masho

**Affiliations:** a Department of Animal Science, Mizan- Tepi University, P.O Box: 260, MizanAman, Ethiopia

**Keywords:** Daily milk yield, Dairy cow, Kaffa, Lactation milk yield, Productive performance

## Abstract

The current research focused on the effects of parity, season, and lactation stage on the milk yield of indigenous dairy cows in selected districts of the Kaffa Zone of southern Ethiopia. The districts of Gesha and Chena were purposefully chosen. The study design for the 384 household surveys was a cross-sectional survey with a simple random sample approach as the sampling method. Following the survey, 192 lactating cows were chosen for a monitored investigation to track nighttime and morning milk supply. In addition to key informants and focus group discussions, primary and secondary data were obtained via a semi-structured questionnaire, interview, and field observation. The most favored features functioning as selection criteria were those favored for production, reproduction, physical appearance, physiological function, and temperament, in that order. Evening milk yield (EMY), mornning milk yield (MMY), daily milk yield (DMY), lactation length (LL) and lactation milk yield (LMY) were 0.91 ± 0.033 L, 1.22 ± 0.037 L, 2.125 ± 0.07 L, 6.36 ± 0.116 months, and 427.10 ± 20.678 L, respectively, with significant difference (p < 0.01) amongst districts, parity, and season. However, there was no significant variation between studies in EMY, MMY and DMY. Significantly higher values of production performances were recorded for interaction among districts, parity, and season. The study also revealed that interaction between district (Gesha) and parity (third), which were signficantlly higher for EMY, MME, DMY, LL, and LMY 1.50 L, 2.00 L, 3.50 L, 8.10 months, and 850.50 L, respectively. The same trend also observed for interaction among district (Gesha), season (wet), and parity (third), which were obtained to be highly significant values of EMY, MMY, DMY, LL, and LMY were 1.35 L, 1.75 L, 3.10 L, 7.65 months, and 716.80 L, respectively. The study region has a large population of indigenous dairy cows and produces higher yields than the national average. However, it falls short of the worldwide production benchmarks, and the trait preferences used as a selection criterion were based on the farmers' indigenous knowledge. Measures to enhance production abilities must be incorporated and selection criteria must be modernized.

## 1 Introduction

Sub-Saharan Africa has a high population of indigenous cattle, with 150 different varieties accounting for 20 % of the global cow population [1]. Ethiopia serves as a crossroads for domestic animals from Asia to Africa, and it now ranks first in Africa and fifth in the globe [2]. Ethiopia has 65.35 million cattle, with female cattle accounting for 55.90 % and male cattle accounting for 44.10 % [3]. The population consists of 98.2% unimproved indigenous breeds, 1.62% cross-breds, and 0.18% foreign pure breeds [4]. According to DAGRIS [5], Ethiopia has 37 recognized indigenous cattle breeds that provide draught power, milk production, meat, and revenue. Cattle are also contributing to society's social and cultural values. Milk intake per capita was 19 kg per year, significantly lower than the African and global averages of 40 kg and 105 kg per year, respectively [3]. Currently, the total milk production from the country's 12.39 million milking cows is projected to be around 3.3 billion liters, with a daily milk yield of 1.5 L and an average lactation time of 6 months [2]. Similarly, the lactation period of indigenous cattle was 9.9 months [6]. Cattle of indigenous breeds produced an average daily milk output of 1.40 [7].

The most prevalent features of favor (preferred) were milk yield and traction power, fertility, body weight, feeding behavior, temperament, color and disease resistance, and hard environmental conditions, which differed according to the production method, cultural tradition, and agro-ecology (G tolerance, and resilience to limited feed availability and water supply) [4]. Kaffa Zone is located in the south western regional (SWR) state. It is the home of 5,218,959 cattle [8]. However, similar to other regions of Ethiopia, livestock production is constrained by different factors, such as insufficient feed supply in terms of quality and quantity, animal diseases, poor management practices, and a lack of improved breeds. The milk production performance of indigenous dairy cows are not studied and documented, except few attemptes of study on dairy cattle management practices and constraints in the zone. Therefore, this study was aimed at evaluating trait preferences and the effects of parity, season, and lactation stage on the production performance of indigenous dairy cows in selected districts of the Kaffa Zone.

## 2 Materials and methods

### 2.1 Description about the study areas

**Fig. 1:**
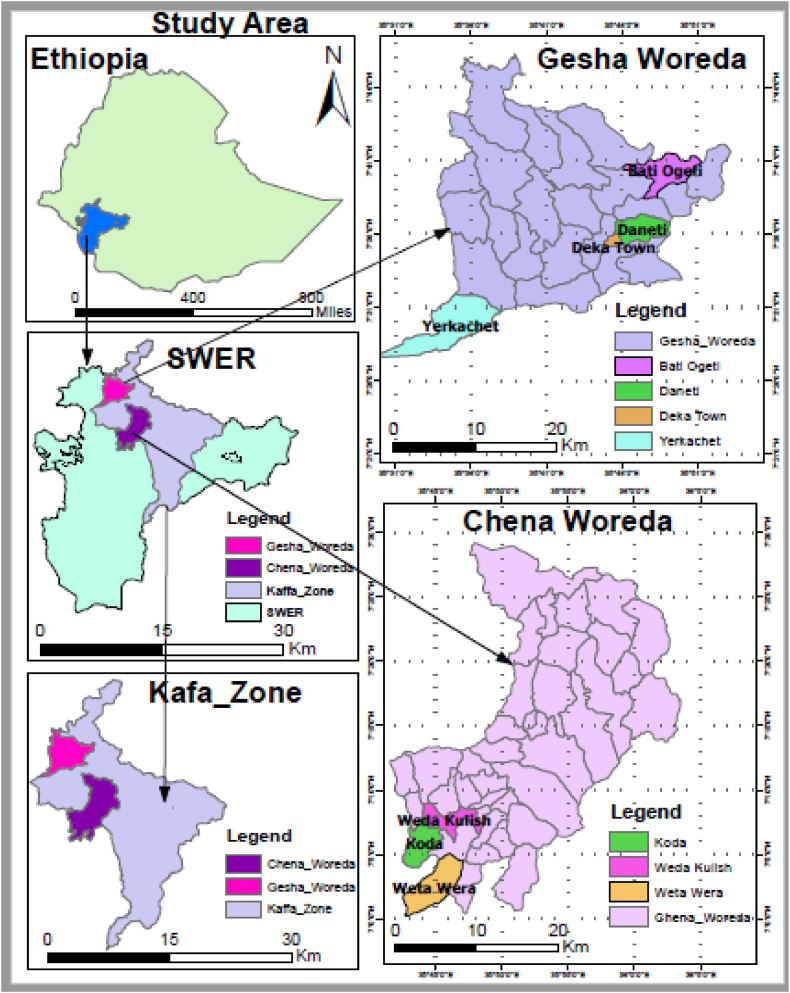
Map of study area.

Kaffa zone located between 6° 24' and 8° 13' north latitudes and 35^0^30' and 36°46' east longitude. It bordered the northern Jimma zone in the north, the Sheka zone in the north-west, Bench-sheko in the south-west, the west Omo zone in the south, and Konta special woreda in the southeast. It is divided into three traditional climatic zones based on height and climatic considerations. The Highland (2500–3000 masl), Midland (1500–2500 masl), and Lowland (500–1500 masl) made up 11.6%, 59.5%, and 28.9 % of the total area, respectively. The yearly temperature ranged from 10.2 to 27.5° Celsius, with rainfall ranging from 100 to 2450 mm. Depending on humidity, the dense rainy season is June–September, the transitional or short rainy season is October, November, and May, and the dry season is December-April [8]. As a result, for the current study, the dry season was represented by April, while the rainy season was represented by June. A mixed crop-livestock production method was used to produce dairy cows [8]. Accordingly, two representative districts namely Geha and Chena were selected to evaluate the effect of parity, season and lactation stage on production performance of indigenous dairy (Fig. 1).

### 2.2 Study design

The types of study designs were both cross sectional study design for surveys and longitudinal study design for follow up (monitored study) of 192 indigenous dairy cows.

### 2.3 Sampling techniques

Aside from the districts that were specifically chosen, three kebeles from each district were picked using basic random selection approaches (six kebeles in total). Similarly, 64 farmers were chosen at random from each of the kebeles for the cross-sectional research (a total of 384). For the monitoring project, 32 lactating cows with varying parity numbers and lactation phases were purposefully selected from survey data. The reasons were twofold: first, to avoid data recording stoppage if a cow became dry; and second, to investigate the effects of an equal number of parities and lactation stages on milk production in the evening, morning, and day milk yields using fixed variables.

### 2.4 Sample size determination

The desired sample size for the study was calculated by the formula given by Thrusfield [9].

Consequently, 95 % confidence interval is required with 5 % margin of error in current study. Hence, sample size (n) is determined by the following formula:

(1) $$\frac{n=\left(Z\alpha^{2*}\left(\text{expected}\;\text{population}\right)*\;\left(1‐\text{expected}\text{population}\right)\right)}{e^{2}}$$

Where, n = required sample size.

Expected population proportion = 50 % (0.5)

e = desired absolute precision or the margin of error 5 % (0.05)

Zα = is the abscissa of a normal curve that cuts of an area at the tails with 95 % confidence interval and the highest margin of error 5 % having 1.96 in Z table

$$\frac{n=\left({Z\alpha }^{2*}\left(\text{expected}\;\text{population}\right)*\;\left(1‐\text{expected}\text{population}\right)\right)}{e^{2}}$$

$$n=\;\frac{{\left(1.96\right)}^{2*}\;{\left(0.5\right)}^{*}\left(1-\;0.5\right)}{0.05^{2}}$$

n=384.16.~n=384

Following the survey, a monitoring study was conducted on 192 lactating indigenous dairy cows. Each district was allocated 96 lactating dairy cow with 32 lactating cows, having different parity and lactation stages in each kebele. For reliable lactation length stratification, survey data was immediately analyzed as soon as it was completed. In this way average lactation length (LL) of indigenous dairy cow in the study area was found 6.36 *±* 0.116 months. Therefore, LL was categorized into three stages: early lactation stage (1–2 months), mid (3–4 months) and late (≥5 months). Moreover, parity number were classified as first, second and third (≥3) parities (parities above three were merged together as third due to their non-significant statistical variation and limited access to higher parity data). Further, for fixed effect of wet and dry seasons of the year, a monitoring study was held for milk yield at evening, morning, and daily milk with intimate follow-up, and lactation milk was calculated by multiplying daily milk yield with lactation length. The amount of milk yield was measured with a volumes calibrated plastic jog.

### 2.5 Sources and methods of data collection

The types of data sources were both primary and secondary. Primary data were collected from all households, focus group discussions (FGD) and key informants through a well-structured and pretested questionnaire, an interview, and field observation. Key informants encompassed livestock and fishery development office heads, private practitioners, traders, and input suppliers. Group discussions were conducted with top kebele administrators, model farmers, and extension workers having 7–10 members in each kebele. Principled enumerators who graduated from TVET and Dandi boru Animal Science College were selected, trained, and employed with the closest researcher following up for significant and relevant data collection. Secondary data were collected from written documents of the livestock and fishery resource development department and offices, agriculture and natural resource development department and offices, the center of AI, epidemiology reports, veterinary anamnesis records, published journals, articles and books.

### 2.6 Data management and analysis

Collected data on livestock population and land holdings of individual farmers were entered into MS Excel spread sheet (Excel, 2010) for data clearance and analysed using stastical analyisis system (SAS) software 9.3 version [10]. Indices were calculated to rank trait preferences of indigenous cows by the farmer according to the following formula: Index and the value of index correspond to order of ranks (the highest index value, the most desired/favored variable). Index is the sum of (n times number of response criteria ranked first + n times number of response ranked second...+ number of response ranked nth) given for particular qualitative variables divided by the sum of responses under each rank summation of (n-1times total response ranked first + n- 1 times total response ranked second...+ total response ranked nth) for all qualitative variables (criteria) considered [11].

(2) $$\text{Index}=\frac{\sum \left(n\;x\;ranked1^{st}\right)+\left(n-1\right)\times ranked2^{nd})+\ldots +1x\;ranked\;last)\;\text{for}\;\text{specific}\;\text{trait}}{\sum \left(n\;x\;ranked1^{st}+\left(n-1\right)\times ranked2^{nd}+\ldots +1x\;ranked\;last\right)\text{for}\;\text{all}\;\text{trait}\;\text{in}\;\text{cotempilation}}$$

Where: n = the number of trait ranks in concern.

Milk production performances such as evening milk yield, morning milk yield, daily milk yield, lactation milk yield, and lactation length were analyzed using general linear model (PROC GLM) procedure of SAS [10]. If there was a significant difference between means, Tukey honestly significance test at α < 0.05 and α < 0.01 were considered as significantly and highly significant difference respectively to adjust the mean separation. The model for the analysis was:Yijklm = μ +ai + bj + ck + dl + ep+(a*b)ij +(a*c)ik+(b*c)jk +(a*b*c) ijk+εijklm. Where; Yijklm = Response variables; μ = the overall mean (intercept); ai = effect of district; bj = effect of parity; cl = effect of season; dl = effect of lactation stage; ep = effect of study; (a*b)ij = interaction between district and parity; (a*c)ik = interaction between district and season; (b*c)jk = interaction between parity and season; (a*b*c) ijk = interaction among district, parity and season; εijkl = random error.

## 3 Results and discussions

### 3.1 Cattle population and land per household

The cattle population and land holdings per household in the study area are presented in Table 1. Cows were a leading members with 3.31 ± 0.12 followed by calves 2.53 ± 0.10, bulls 1.05 ± 0.01, heifers 0.77 ± 0.03, and steers 0.26 ± 0.01 without significant variation between districts. Fissha and Deng [12] reported a little bit different result verifying cows 4.75 ± 0.19, heifers 1.76 ± 0.13, bulls1.68 ± 0.11, and calves 1.65 ± 0.07 and steers 1.07 ± 0.09 in lowland parts of Ethiopia. Abebe et al. [13] reported, average dairy cow 4.48 ± 1.85, heifers 1.89 ± 1.0 and 1.66 ± 0.47 bulls. Lower cattle population as calves 1.28 ± 0.04, heifer 1.39 ± 0.04, bull 1.35 ± 0.05, and lactating dairy cow 1.25 ± 0.04 were reported in Jimma zone dairy producers [14]. Land holdings per individual household was 2.72 ± 0.40 ha, with a significant difference (p˂0.05) between districts. It was in agreement with Yaynshet et al. [15] but higher than Abebe et al. [13]. Dairy production system, prevalence of epidemic and endemic diseases, accessibility of land, purpose of production, and agro-ecological factors were responsible for livestock and dairy cow population variation.

**Table 1:** Livestock population and Household land holding capacities.

Livestock species	Gesha (Mean ± SE)	Chena (Mean ± SE)	Overall (Mean ± SE)
Land holding (Hec)	2.93 ± 0.18	2.51 ± 0.62	2.72 ± 0.40
Cattle population			
Cows	3.52 ± 0.10	3.10 ± 0.02	3.31 ± 0.12
Calves	2.74 ± 0.03	2.32 ± 0.10	2.53 ± 0.10
Bulls	1.10 ± 0.07	1.00 ± 0.04	1.05 ± 0.01
Heifers	0.83 ± 0.01	0.71 ± 0.02	0.77 ± 0.03
Steers	0.33 ± 0.01	0.20 ± 0.00	0.26 ± 0.01

SE: standard error, NA: not available, **: p˂0.01, *: p˂0.05, NS: non-significant (p˃0.05), Hec: Hectare.

### 3.2 Dairy cow management practices

#### 3.2.1 Feed types and sources

The feed type and sources such as grazing land, supplementary and complementary feed are presented in Table 2. This study revealed, mixed grazing style (grazing both on communal land and private land) was predominant accounting for 57.81 % followed by 35.94 % private land grazing and 6.25 % grazing only on communal land with significant variation (p˂0.05) between districts. Similarly, Abebe et al. [13] reported that crop residue/hay, concentrate feeds, industrial by products (oil cake and wheat bran), and local beverage products (Attela) were a common feed sources used by dairy producers. Besides, Tassew [16] from Bahir Dar, Tsadikan [17] from Enderta district, Tigray regional state and Gezu et al. [18] from Hossana reported that industrial byproducts, crop residues, and hay, were the major feed resources for dairy cow which was dissimilar with dairy cow feed sources of current study. Zero grazing (63 %) but rotational grazing (14 %) and free grazing (13 %) was reported [19]. Girma et al. [20] study revealed, in dairy production system due to shortage of a grazing land most farmers feed their dairy cattle by cutting green feeds, crop residues, atela and mill byproduct which is consistent with the result of current study. Most probably, this was management system variation and adoption of local dairy cows for free grazing. Moreover, the inaccessibility of concentrates due to infrastructural constraints can led farmers to intimate to only natural way of grazing.

**Table 2:** Feed type and sources of dairy cow.

Grazing land	Gesha	Chena	Total average	Chi-square	P value
Communal	(15) 7.8 %	(9) 4.7 %	24 (6.25 %)	7.133	0.028 (*)
Private	(57) 29.7 %	(81) 42.2 %	138 (35.4 %)		
Mixed	(120) 62.5 %	(102) 53.1 %	222 (57.81)		
Supplementary and Complementary Feeds					
LL	5.20 %	10.90 %	8.10 %	11.105	0.025 (*)
LLA	6.30 %	13.00 %	9.60 %		
LLINCMVA	47.40 %	41.10 %	44.30 %		
MV	6.30 %	7.30 %	6.80 %		
NCMV	34.90 %	27.60 %	31.30 %		

LL: leaves and leftovers; LLA: leaves, leftovers and aftermaths; LLINCMVA: leaves, leftovers, improved forages, non-conventional feed, concentrates, minerals, vitamins and aftermath. MV: minerals and vitamins; NCMV: non-conventional feeds, minerals, and vitamins; significantly different at * p˂0.05.

#### 3.2.2 Housing dairy animals

Complete house enclosure (24 %), tethering at nearby home (46.1 %), and extensive layover (29.95 %) were mechanisms by which farmers kept their dairy cows at night in worse climatic condition with no significant variation (p˃0.05) between districts of current study area (Table 3). Calves stay at home together with farmers for one month and allowed to dwell outside enclosure or veranda. This result inconsistent with Lenco and Seblewongel [21] with (100 %) using separate houses for keeping the animals, most of the cows (93 %) housed in concrete type barn, and 6 % were in muddy soil floor and only 1 % in wooden floor. Similarly, 68.3 % barn, 23.3 % open shed and 8.3 % with no house access in lowland area of Ethiopia [12]. Similarly, Bruktawit [22] reported most farmers used barn for their dairy cow. Farmers (100 %) use separate house for keeping dairy cow and most of the cows (93 %) were housed in concrete type floor barn [21]. Abebe et al. [13] reported Separate/loose house (61 %) and mixed with households (39 %) disagreed withy current study result. Dachu and Kefele [19] reported that majority of the farmers were housed their cattle separately from family house 67 %, whereas 33 % of the farmers were house their cattle together with family house all standing at extreme edge with reference to current study result. This was due to poor management practices and ill awareness of farmers about welfare of dairy cows.

**Table 3:** Pattern of housing, frequency of cleaning and floor types.

Variables	Gesha (n = 192)		Chena (n = 192)		Total	
	Frequency	%	Frequency	%	%	P value
Condition of shelter						0.121(NS)
Complete house	40	20.8	52	27.1	24	
Tether/paddock	86	44.8	91	47.4	46.1	
Extensive lay over	66	34.4	49	25.5	29.95	
Roof type						0.344(NS)
Corrugated iron	9	4.7	13	6.8	5.73	
Grass	31	16.1	39	20.3	18.23	
Floor type						0.354(NS)
compact soil	32	16.7	41	21.3	19.01	
Wood products	8	4.2	11	5.7	4.95	
Cleaning schedule						0.405(NS)
Once	17	8.9	14	7.3	8.1	
Twice	9	4.7	16	8.3	6.5	
Conditionally	14	7.3	10	5.2	6.25	

Significantly different at * p˂0.05.

According to the key informants, survey responses, and FGD, tethering was to avoid crop damage, to prevent predatory attacks, Proper utilization of natural resources, prevent unwanted breeding and to collect dung for natural fertilizer. The roofs were constructed from corrugated iron and local grass are 5.73 %, and 18.23 %, respectively. Floors made from compact soil and wood products like bamboo tree and timber 19.01 %, 4.95 % with no significant variation (p˃0.05). Cleaning was once (8.1 %), twice a day (6.5 %), and conditionally depending up on accumulation of dirty, mud, and muck accounted (6.25 %) in study area with no significant difference (p˃0.05). Conversily, the majority of farmers (70 %) cleaned dung and urine from dairy cattle house twice a day in morning and evening while (30 %) cleaned it only once per day in morning [19]. House sanitation is necessary to have health that enables to control and prevent different transmissible and contamination disease and parasites, which cause to sick, reduce production and reproduction performance of dairy cattle. As other management drawback, animal welfare neglectance is responsible for occurrence of housing and cleaning variation in study area.

#### 3.2.3 Watering frequencies and water sources

Watering frequencies and water sources of the current study are presented in Table 4. The investigation revealed that no availability of pure water supplies not only for animal watering but also for human uses. Most sources of water for home consumption were from groundwater constructed for communal use besides of borehole and springs. This indicated how much infrastructural constraints had been dominating the study area yet. Sources of water were rivers (35.5 %), wells (13.25 %) and ponds (51.55 %) in the dry season and rivers (45.3 %), wells (35.15 %) and ponds (19.55 %) in wet season of the year with highly significant difference (p˂0.01). This result disagreed with source of water for dairy cows in Gambella region of rivers (47.5 %) and ponds and wells (20.0 %) [20]. Dachu and Kefele [19] reported that only the two source of water namely river (70 %) and pond (30 %) covered water demand of dairy cow in aldadaworeda. Bruktawit [22] reported that 98.9 % used pipe water and the other 1.1 % use well water. Similarly, 98 % used pipe water as thier main water sources and the other 2 % used well water [20].

**Table 4:** Water sources and watering frequency of dairy cow.

Water sources	Gesha (n = 192)		Chena (n = 192)		Total average		Chi-square	P value
	DS	WS	DS	WS	DS	WS		
Rivers	37.50 %	47.40 %	32.80 %	43.20 %	35.5	45.3	71.6	0.00
Well	16.10 %	33.30 %	10.40 %	37.00 %	13.25	35.15		
Pond	46.30 %	19.30 %	56.80 %	19.80 %	51.55	19.55		
Watering frequency								
Once	14.10 %	56.30 %	7.80 %	47.90 %	10.95	52.1	8.52	0.001
Twice	65.60 %	34.00 %	74.50 %	39.60 %	70.05	36.8		
Three	20.30 %	6.80 %	17.70 %	12.50 %	19	9.65		

DS = Dry season; WS= Wet season; significantly different at * p˂0.05.

Moreover, river 30.3 %, well 24.6 %, supplied pipe/tank 40.5 %, and spring water 4.5 % was reported [12]. Watering frequencies were found once at 10.95 %, twice at 70.05 %, and three time at19 % in the dry season of the year and once at 52.1 %, twice at 36.8 %, and three time at 9.65 % in the wet season of the in study area with highly significant variation (p˂0.01). This is contrary to 55 % and 45 % free access, 26.7 % and 21.7 % twice a day, and 18.3 % and 33.3 % in pastoral and mixed crop production in the study districts [12]. Watering once a day (16 %), twice a day (47 %), and free access 37 % was reported for dairy cow [12].

#### 3.2.4 Health care and prevalence of endemic dairy cow diseases

**Fig. 2:**
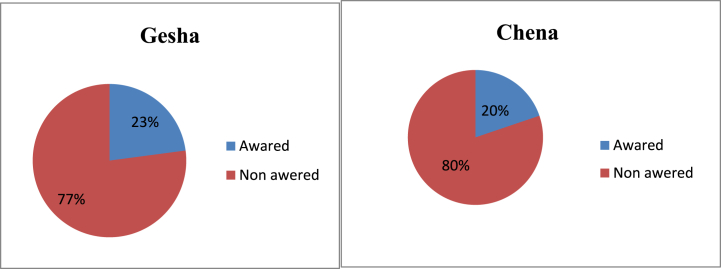
Awerness of the farmers in the study area toward endemic dairy cow diseases.

Awareness of the farmer about common zoonotic diseases (Fig. 2) and the prevalence of endemic dairy cow diseases of the current study are presented in Table 5. Emblematically, farmers consider as cow diseases caused by a few pathogenic agents and commonly called Gogishoo (native language). Relatively and trivial proportions about 77 % and 80 % were unfamiliar with the concepts and believed any disease as curse of God or Allah and evil spirit attack and 23 % and 20 % were intimate with zoonosis for Gesha and Chena, respectively (see Fig. 2).

**Table 5:** Prevalence of endemic dairy cow diseases.

Diseases	Gesha (192)		Chena (192)		Average
	Frequency	Percentage	Frequency	Percentage	Percentage
Mastitis	29	15.1	32	16.7	15.88
Abortion	14	7.3	11	5.7	6.5
Rabies	3	1.6	1	0.5	1.04
FMD	7	3.6	9	4.7	4.17
BMCF	9	4.7	6	3.1	3.9
Salmonellosis	21	10.9	24	12.5	11.72
Pasteurellosis	15	7.8	13	6.8	7.29
CBPP	5	2.6	7	3.6	3.12
Brucellosis	9	4.7	6	3.1	3.9
Black leg	5	2.6	8	4.2	3.38
IBK	6	3.1	3	1.7	2.34
BTB	2	1	5	2.6	1.82
Anthrax	1	0.5	0	0	0.26
Ticks	30	15.6	36	18.8	17.19
Lice	11	5.7	9	4.7	5.21
Mite	4	2.1	1	0.5	1.3
Nematode family	6	3.1	2	1	2.08
Trematode family	10	5.2	16	8.3	6.77
Cestode family	5	2.6	3	1.6	2.1

NB. The current study targeted on only infectious diseases. Here, surgical causes, non-infectious and metabolic disorder were neglected. The reason was to pave a way for individual, regional, and national institution researchers for vaccine preparation and eradication of diseases due to their zoonosis. Moreover, it alarms and awake concerned bodies to resolve any of constraints investigated in current study and further more.

Similarly, Lenco and Seblewongel [21] reported that awareness of farmers was limited to specific diseases like tuberculosis (38.89 %), mastitis (33.33 %), anthrax (19.84 %), brucellosis (6.35 %), and salmonellosis (1.6 %). These authors also reported awareness levels of milk-borne zoonosis in farmers below 50 %. Secondary data were obtained from epidemiology reports, veterinarians, and anamnesis books of selected kebeles and woreda livestock and fishery resource development offices to make the wisest discoveries about frequently prevailing endemic diseases. For the taxonomy, diseases were categorized under 1st multi-factorial, those can be caused by various etiological agents; 2nd viral diseases; 3rd bacterial diseases, and 4th parasitic diseases. This study revealed and declared Ticks, 17.19 %, Mastitis, 15.88 %, Salmenelosis, 11.72 %, Pastuerella, 7.29 %, Trematodes, 6.77 %, Abortion, 6.5 %, Lice, 5.21 %, FMD, 4.17 %, Brucellosis, 3.9 % and BMCF, 3.9 % were top ten endemic diseases. Tesfamicheal and Yien [12] reported, slightly divergent result as Trypanosomiasis, Pastuerllosis, CBPP, FMD and Internal and external parasites were only top five diseases frequently had been occurring (see Table 6).

**Table 6:** Traits of preferences for indigenous dairy cow.

	Gesha (n = 192)			Chena (n = 192)		Total
Traits	Parameters	Index	Rank	Index	Rank	Index (Rank)
Production						0.309/1st
	Milk Yield	0.468	2nd	0.465	2nd	
	Traction	0.532	1st	0.535	1st	
Reproduction						0.258/2nd
	Short AFC	0.269	2nd	0.139	4th	
	Short CI	0.229	3rd	0.244	2nd	
	LRLS	0.313	1st	0.282	1st	
	LBS	0.188	4th	0.156	3rd	
Physiological						0.120/4th
	Disease Resistance	0.394	1st	0.27	3rd	
	ST	0.263	3rd	0.385	1st	
	FCE	0.34	2nd	0.345	2nd	
Morphological						0.226/3rd
	Horn	0.106	6th	0.07	6th	
	Hump	0.123	4th	0.149	3rd	
	SS	0.178	1st	0.204	1st	
	LULT	0.123	4th	0.139	5th	
	LMC	0.137	3rd	0.143	4th	
	Red color	0.161	2nd	0.151	2nd	
	White color	0.039	9th	0.05	8th	
	Black color	0.07	7th	0.03	9th	
	Bronze color	0.06	8th	0.066	7th	
Temperament						0.087/5th
	Docility	0.563	1st	0.475	2nd	
	Adaptability	0.437	2nd	0.525	1st	

**Indication**: AFC: Age at first calving, CI: Calving interval; FCE: Feed conversion efficiency LRLS: Longer reproduction life span, LBS: Large body size, SS: Structural soundness, LULT: Lager udder and longer teat, LML: Larger mouth circumference.

### 3.3 Preferred traits for indigenous dairy cow

Trait preferences of dairy cows in the study area were presented in Table [6] according to their decreasing ranks of favor (traits of production, traits of reproduction, traits for morphological appearance, traits for physiological function, and traits for temperament). Production traits such as milk yield and milk composition were favored as first and second ranks, respectively. From traits of reproduction, age at first calving, age at first service, service per conception, and age at first puberty were ranked in descending order. From a physiological standpint, feed intake, body weight gain, and udder health were favored. Traits of temperament, cow comfort to manage, and feeding cost were highly desired traits [21].

The study revealed that, high milk yield, high reproduction potential, traction power, high live weight, stress tolerance, feed conversion efficiency, and red coat color were preferred traits in descending order [12]. Afras [23] reported, income generation, milk production, good mothering ability, shortening of calving intervals, traction, meat production and source of breeding bulls were preferred traits. Similarly, Misganaw et al. [24] and Chawala et al. [25] reported disagreable results.

Besides milk production, traction power, adaptation, and disease resistance ([[26], [27], [28], [29]]); growth rate and fertility [30]; body conformation and coat clour [31] are preferred traits for cattle production. Generally, CSA [4] reported that tropical cattle are endowed with traits of adaptability to harsh environmental conditions, disease resistance, heat tolerance, resistance to low feed and water supplies and poor management system. The result of the current study results revealed that traits are inconsistent depending on individual perception, agro-ecological variation, socio-cultural traditions, breeding objectives and production system as it was reported by Ref. [23].

### 3.4 Effect of district, parity, season, lactation stage and study on production performance

#### 3.4.1 Effect of districts on production performances

Climatic factors are totipotent in the lives of organisms all over the world. Production performances of dairy cows in the study area were presented in Table 7. Here it was found that, milk yield obtained in the present study at evening (1.001), morning (1.34), and daily (2.34) lactation milk yield (494.40) liters was signficantly (p˂0.01) higher for Gesha district as compared to evening (0.82), morning (1.103), daily (1.93), and lactation milk yield (360.33) liters obtained from Chena District. Similarly, significantly (p˂0.01) higher values of lactation length also observed for Gesha (6.77 months) district as compared to Chena (5.96 months) district. The differences in milk yield and lactation length maight be attributed to the availability of animal feed in Gesha Districts. However, yields recorded from both districts were higher than the values of 0.73 ± 0.02 and 0.99 ± 0.02 L reported for local cows in the Guraghe zone [32]. George et al. [33] also reported the same logic for significant variation for evening milk yield of 109.67 ± 7.66 L and morning milk 170.27 ± 5.51 L at herd level with highly significant variation. Current study publicize, higher DMY than the value of 1.40 ± 0.05 L reported by Yeneshet et al. [15] in the northern Gondar zone. Moreover, it was greater than 1.37 L at the national level [4], 1.54 L in Belay [34] and 2.0 L in Bainesagn [35]. Similarly, Beriso et al. [36] reported 1.45 L in Chuko district in South Nation Nationality Peoples Region, 1.52 ± 0.86 L of Hundie et al. [37] in Guduru livestock production and research center, 1.32 ± 0.11 L of Angot district in wollo [38]. The current findings was also lower than those of Sheko breed (2.79 ± 0.06 L) Bayou et al. [39], Begait breed (2.7 ± 0.3 L) Teweldemedhn [40] and Fogera breed (3.54 ± 0.14 L) Kebede et al. [41].

**Table 7:** Effect of District, Parity, Season, Lactation Stage and study on Production Performance of indigenous cow.

District	EMY(LSM) liter	MMY(LSM) liter	DMY(LSM) Liter	LMY(LSM) liter	LL (LSM)month
Chena	0.820^b^	1.103^b^	1.93^b^	360.33^b^	5.96^b^
Gesha	1.001^a^	1.340^a^	2.34^a^	494.40^a^	6.77^a^
Average	0.91	1.22	2.135	427.365	6.365
SEM	0.8	0.004	0.006	2.03	0.01
Parity number					
First	0.60^a^	0.84^a^	1.44^a^	237.32^a^	5.18^a^
Second	0.94^b^	1.19^b^	2.13^b^	416.27^b^	6.63^b^
Third	1.20^c^	1.64^c^	2.83^c^	628.52^c^	7.28^c^
Average	0.91	1.22	2.13	427.37	6.36
SEM	0.0024	0.005	0.006	3.5	0.02
Season					
Dry	0.84^b^	1.102^b^	1.85^b^	337.17^b^	5.73^b^
Wet	0.97^a^	1.42^a^	2.41^a^	517.60^a^	7.00^a^
Average	0.91	1.26	2.13	427.39	6.37
SEM	0.003	0.01	0.013	4.55	0.022
Lactation Stage					
Early lactation	0.84^c^	1.34^b^	2.18^b^	447.92^b^	6.41
Mid lactation	1.00^a^	1.69^a^	2.67^a^	454.00^a^	6.3
Late lactation	0.90^b^	1.14^c^	2.03^c^	382.84^c^	6.36
Average	0.913	1.39	2.293	428.25	6.36
SEM	0.0024	0.011	0.018	4.4	0.03
Study					
Survey	0.92	1.224	2.14	427.36	6.36
Monitoring	0.91	1.227	2.13	429.15	6.36
Average	0.915	1.2255	2.135	428.3	6.36
SEM	0.002	0.0004	0.006	1.8	0.012
P-value					
District	<0.001	<0.001	<0.001	<0.001	<0.001
Parity number	<0.001	<0.001	<0.001	<0.001	<0.001
Season	<0.001	<0.001	<0.001	<0.001	<0.001
Lactation Stage	<0.001	<0.001	<0.001	<0.001	0.3
Study	0.14	0.15	0.14	0.5	0.9

^a-c^ Least square means bearing different superscripts within factor across column are significantly different at * p˂0.05 and **p˂0.01; SEM=Standard Error Mean; EMY; Evening milk yield; MMY:Morning milk yield; DMY: Daily milk yield; LMY: Lactation milk yield; LL: Lactation length; LSM: Least square mean.

The values of lactation milk yield (LMY) obtained in the current study were higher than 225 L of Danakil breed [5], 203.54 ± 1.40 L of Smada breed [42] and 339.2 L of [8]. However, LMY was lower compared to tropical and sub-tropical areas of Jersey breeds and Crossbred HF (4738.28) liters [43]. The value of lactation length (LL) obtained in the current study was comparably higher than 6.0 months LL at the national level [4]. Contrarily, the current result was lower than 6.78 months of Simada breed in south Gondar [42]. Besides, 8.2 months of Kumar et al. [44] in Dandi district, 9.93 ± 0.2 months of Beriso et al. [36] in Chuko district, 8.08 ± 0.12 months of [8], and 9.9 months in north Gondar town [6]. The result of current study ratify that, environmental factors can determine production performances of the same breed. Moreover, environment, genome, and environment-genome interaction were appreciated between different breeds of the same species of dairy cows.

#### 3.4.2 Effects of parity on production performances

Stages of maternity (parity) had an effect on the production performance of dairy cows (Table 7). The present study found that there was a highly significant difference (p˂0.01) between parity numbers in evening milk yield (EMY), morning milk yield (MMY), daily milk yield (DMY), lactation milk yield (LMY), and lactation length (LL). Current results publicize that milk yield increased in parallel fashion with parity numbers. Contrarily, Khansefid [45] reported that parity of the dam only had a limited effect on first lactation milk yield. According to Astiz et al. [46], no difference was reported in performance between parity numbers. Similarly, no parity effect on milk yield was reported [47]. In the first, second, and third parities, 1.44 L, 2.13 L, and 2.83 L of DMY were found to have a highly significant differences (p˂0.01) in this study. Nicolas et al. [48] narrated an agreeable result by reporting the direct proportionality of milk yield and parity number. Contrary to the current results, Mohamed et al. [49] verified that parity numbers had no impact on production performances. In their report, scattered DMY with first, second, third, and fourth parity for exotic dairy cow were indicated 26.54 ± 0.14, 27.09 ± 0.15, 26.70 ± 0.21, and 25.55 ± 0.25, respectively, without a significance variation. In reverse concept, 4659.28 and 4817.27 L of LMY in first and second parity crossbred Holstein Friesian without significant variation (p˃0.05) in different parts of Ethiopia were reported, which was different from the current study result [50]. In this study, similar growth of party number and LL was appreciated, and Kakati et al. [51] reported an agreeable concept. Yeneshet et al. [15], reported a higher 8.08 ± 0.12 months LL but similar logic to current study for 3–5 years, 6–9 years and >10 years old indigenous dairy cows having 7.73 ± 0.13, 7.76 ± 0.10, and 8.76 ± 0.13 months, respectively. Kashoma et al. [50] reported that, primiparous cows had significantly longer lactation lengths than multiparous cows contrary to the current study. Senbeta and Abebe [52], reported 322.38 days and 304.08 days LL for first and second parity with no significant difference. According to current study, the most probable source of variation with parity were advancement of mammary gland secretory cells, gynecological organ and system maturity and efficient hormonal regulation as parity number goes up.

#### 3.4.3 Effects of season of the year on production performances

Table [7] demonstrates that, how much season of the year determines production capacity of dairy cows. Evening milk yield, morning milk yield, daily milk yield (DMY), laction milk yield (LMY), and lactation length (LL) were greatly affected (p˂0.01). This diue to climatic factor (season of the year) of longer dry season can trigger mammary gland involution accompanied by apoptosis and autophagy, resulting in a decreased amount of mammary epithelial cells that ultimately cause a decline in milk yield, as it was reported by Prathap et al. [53]. Liu et al. [54] narrated, elevated temperature and humidity negatively affect feed intake, affecting the reproductive potential, which ultimately decreases milk production. Mohamed et al. [49] conducted offensive results with non-significant variation between the winter (27.20 L) and summer (26.60 L) DMY of Holstein and Brown Swiss in Egypt. Baset et al. [55] narrated an agreeable result on the effects of season on lactation milk yield. The current study result was higher and contradictory to 223.68 ± 3.55 L and 229.32 ± 1.58 L in the wet and dry seasons of the year without a significance difference [56]. Habibi et al. [57] reported a higher LMY of a Holstein Friesian cow of 17,060.85 ± 34.5 L in summer and 16,398.45 ± 31.5 L in winter as a friendly concept. Contrary to the current result, Darfour-Oduro et al. [58] reported non-significant effects of season on LL for Sanga-Holstein Friesian crosses in Ghana with dissimilar results. As an agreeable concept, season of calving had a significant influence (p < 0.05) on lactation length of Ankole breed cows in Rwanda [59]. The result indicated that thev production performance of indigenous dairy cows can be inhibited or accelerated depending on humidity and rainfall. Wet seasons are endowed with green forages, enough grasses, and accessible water sources. This availability of feed and abrupt scarcity during dry season perfectly affect production performances.

#### 3.4.4 Effect of lactation stage on production performances

Table [7] illustrates that lactation stages of dairy cows had a higher influence on milk yield at evening, morning, DMY, and LMY, with a highly significant difference (p˂0.01). The daily milk yield (DMY) obtained was 2.18, 2.67 and 2.03 L for the early, mid, and late lactation stages, respectively, with a significant variation between the first, second, and third lactation stages. Lactation stage significantly influences milk yield at evening, morning, DMY, and LMY. Milk yield increased gradually from the early to mid-lactation phase then decline gradually to the late lactation phase. This was due to physiological restoration and compensatory stage rather than production task in the early lactation stage [60]. At the same time, their mammary gland and vein are not well developed at an early stage, followed by distorted energy durining dystocia and delivery [60]. Fissha and Deng [12] conducted offensive results of 2.0 ± 0.06 L, 3.23 ± 0.07 L, and 1.72 ± 0.06 L in the Gambella region. Demeke [38] reported the effects of lactation on DMY antagonistically. He testified that lactation phase increased, yield would decrease by 1.75 ± 0.12 L, 1.23 ± 0.10 L, and 0.98 ± 0.09 L. The direct proportionality of DMY and lactation stage was determined (12.04 ± 0.7, 13.24 ± 0.8, 14.10 ± 0.6 L) by Habibi et al. [57]. The effects of lactation stage on yield were due to compensatory restoration of exhausted energy during gestation and parturition at the early stage and period of the preparation for the conceived fetus at late stage. Bhardwaj et al. [61] reported the mean average DMY at early, mid, and late lactation stages were 2.0 ± 0.08 L, 2.99 ± 0.08 L, and 1.84 ± 0.09 L, respectively, which was dissimilar to the current study result. Furthermore, DMY at early, mid, and late lactation stages were 1.99 ± 0.08 L, 3.46 ± 0.10 L and 1.57 ± 0.084 L [62].

#### 3.4.5 Effect of study on production performances

The effect of the study on production performances like milk yield at evening, morning, and average (DMY), LMY, and LL presented in Table 7. The results revealed that there were no significant (p > 0.05) differences between the survey and the monitoring study. The monitored study result had a precise value with an estimation survey result of 2.125 ± 0.07 with non significant mean variation. Both monitored and cross sectional survey studies were interdependent (sociable). The close proximity of results was due to the prior well-orientation of the researcher, intimate FGD, and deep awareness creation about the objectives. In all conditions, the amount of milk harvested at the morning was higher than the milk yield at the evening. This was most probably due to the longer hours of night **(14 h) than day length (10 h) in the cow milking trend in the study area. Besides, the physiological rest of various systems in the dairy cow and complete access to ATP that enhance mammary** gland lactogenesis at night are more likely to occur at night than at daytime.

#### 3.4.6 Effect of interaction on production performance of indigenous dairy cow

Districts, parity, and season interact among themselves for evening milk yield, morning milk yield, daily milk yield, lactation milk yield and lactation length (Table 8). A similar study was reported by shibru et al. [56], in which the interaction of fixed factors influences the milk yield and compositions. In Yang et al. [63], parity and season showed a higly significant effect on milk yield, milk lactose percentage, and milk production. The interaction among district, parity, and season revealed that the fluctuation of milk yield in different locations and different seasons differed due to variations in the type and digestibility of forages. The availability and quality of pasture in different districts and different seasons change the availability of nutrients for indigenous dairy cows, which affect milk yield in various stages of lactation. Thus, the interaction effect of district, parity, and season on milk yield and lactation length is noticeable.

**Table 8:** Effect of interaction among the district, parity and season on production performance of indigenous dairy cow.

Interaction			EMY(LSM)	MMY(LSM)	DMY(LSM)	LMY(LSM)	LL (LSM)
Chena	First	Dry	0.40^h^	0.70^f^	1.10^i^	138.60^l^	4.20^k^
Chena	First	Wet	0.60^f^	0.90^f^	1.51^g^	232.69^j^	5.14^i^
Chena	Second	Dry	0.70^e^	1.00^e^	1.70^f^	300.90^h^	5.90^h^
Chena	Second	Wet	1.00^c^	1.20^d^	2.20^d^	448.80^e^	6.80^d^
Chena	Third	Dry	1.00^c^	1.20^d^	2.20^d^	402.60^f^	6.10^g^
Chena	Third	Wet	1.20^b^	1.60^b^	2.80^b^	638.40^b^	7.60^b^
Gesha	First	Dry	0.50^g^	0.80^f^	1.30^h^	195.00^k^	5.00^j^
Gesha	First	Wet	0.70^e^	1.00^e^	1.70^f^	331.50^h^	6.50^e^
Gesha	Second	Dry	0.90^b^	1.20^d^	2.10^e^	390.60^i^	6.20^f^
Gesha	Second	Wet	1.20^b^	1.50^c^	2.70^c^	615.60^c^	7.60^b^
Gesha	Third	Dry	1.20^b^	1.50^c^	2.70^c^	583.20^d^	7.20^c^
Gesha	Third	Wet	1.50^a^	2.00^a^	3.50^a^	850.50^a^	8.10^a^
Average			0.91	1.22	2.13	427.37	6.36
SEM			0.01	0.001	0.002	0.92	0.013
P-value			<0.001	<0.001	<0.001	<0.001	<0.001
Chena	First		0.50^f^	0.80^f^	1.30^f^	185.64^f^	4.67^f^
Chena	Second		0.85^d^	1.10^d^	1.95^d^	374.85^d^	6.35^d^
Chena	Third		1.10^b^	1.40^b^	2.50^b^	520.50^b^	6.85^c^
Gesha	First		0.60^e^	0.90^e^	1.50^e^	263.25^e^	5.75^e^
Gesha	Second		1.05^c^	1.35^c^	2.40^c^	503.10^c^	6.90^b^
Gesha	Third		1.35^a^	1.75^a^	3.10^a^	716.85^a^	7.65^a^
Average			0.91	1.22	2.13	427.37	6.36
SEM			0.001	0.001	0.001	0.65	0.001
P-value			<0.001	<0.001	<0.001	<0.001	<0.001
Chena	Dry		0.70d	0.97d	1.67^d^	280.70^d^	5.40^d^
Chena	Wet		0.93b	1.23b	2.17^b^	439.96^b^	6.51^b^
Gesha	Dry		0.87c	1.17c	2.03^c^	389.60^c^	6.13^c^
Gesha	Wet		1.13a	1.50a	2.63^a^	599.20^a^	7.40^s^
Average			0.91	1.22	2.13	427.37	6.36
SEM			0.001	0.001	0.001	0.53	0.007
P-value			<0.001	<0.001	<0.001	<0.001	<0.001
First	Dry		0.45e	0.75f	1.20^e^	166.80^f^	4.60^f^
First	Wet		0.65d	0.95e	1.60^d^	282.09^e^	5.82^e^
Second	Dry		0.80c	1.10d	1.90^c^	345.75^d^	6.05^d^
Second	Wet		1.10b	1.35b	2.45^b^	532.20^b^	7.20^b^
Third	Dry		1.10b	1.35b	2.45^b^	492.90^c^	6.65^c^
Third	Wet		1.35a	1.80a	3.15^a^	744.45^a^	7.85^a^
Average			0.91	1.22	2.13	427.37	6.36
SEM			0.001	0.001	0.001	0.65	0.01
P-value			<0.001	<0.001	<0.001	<0.001	0.001

^a-l^ Least square means bearing different superscripts within factor across column are significantly different at * p˂0.05 and **p˂0.01; SEM = Standard Error Mean; EMY; Evening milk yield; MMY:Morning milk yield; DMY: Daily milk yield; LMY: lactation milk yield; LL: lactation length; LSM: Least square mean.

#### 3.4.7 Correlation between parity, lactation stage and production performances

Correlations among different variables were verified in Table 9. The strong positive correlation between milk yield at evening (MYE) and milk yield at morning (MYM) (r = 0.98), DMY and evening (r = 0.994) and morning milk (r = 0.986), and parity and DMY (r = 0.831), evening (r = 0.849) and morning milk yield (r = 0.806). There was a strog positive correlation between lactation milk yield (LMY) and evening milk yield (EMY) (r = 0.98), lactation milk yield (LMY) and morning milk yield (MMY) (r = 0.99), lactation milk yield (LMY) and daily milk yield (DMY) (r = 0.99). Parity number had strong positive correlation with evening Milk yiel (r = 0.86), morning milk yield (r = 0.82), daily milk yield (r = 0.84) and lactation milk yield (r = 0.80). Lactation length had a strong positive correlation with parity number (r = 0.74), evening milk yield (r = 0.95), morning milk yiel (r = 0.93), daily milk yield (0.94) and lactation milk yield (r = 0.95). Lactation stages of dairy cows had a moderately positive correlation (r˂0.5) with statistically significant difference (p˂0.01) in all condition of relationship.

**Table 9:** Correlation between parity, lactation stages, EMY, MMY, DMY, LMY and LL.

Correlation	Parity number	Lactation stage	EMY	MMY	DMY	LMY	LL
Parity number	1						
Lactation stage	0.250*	1					
EMY	0.861**	0.319**	1				
MMY	0.822**	0.255*	0.982**	1			
DMY	0.844**	0.286**	0.995**	0.996**	1		
LMY	0.800**	0.264**	0.983**	0.996**	0.994**	1	
LL	0.738**	0.324**	0.947**	0.934**	0.944**	0.955**	1

Significantly different at * p˂0.05 and **p˂0.01; EMY; Evening milk yield; MMY: Morning milk yield; DMY: Daily milk yield; LMY: lactation milk yield; LL: lactation length.

#### 3.4.8 Role of milk, milking hygiene and sanitation

Table 10 presents that the role of milk, milking hygiene, and sanitation of the study area. The study revealed that milk is an ever-fitting and complete food and used for aesthetic (cosmetic value) (17.2 %), economic source (28.6 %), medication especially for the migraines and fracture, in appetency (18.2 %), and home consumption (35.9 %), with no significant variation between districts. Comparably, Gaurav et al. [64] reported that milk contains 29.36 % fat, 26.98 % protein, 38.1 % lactose, and 5.56 % ash used for growth, body functioning, and supportive therapy for malnutrition. Similarly, milk and its byproducts are used for the medication of Hypolipidemia, hypocholesterolemia, hypogonadism, progressive cognitive and memory decline, mineral and vitamin deficiencies, peripheral neuropathy, and multiple neuropsychiatric syndromes [65]. Cleaning of the milking parlor/shed, milking equipment, teat and udder of dairy cow were found once at 68.8 %, twice at 18.5 % (both before and after milking), and conditionally not in regular rhythm were found at12.8 % in the study area with no significant difference. Antagonistic results were reported by Duguma and Geert [66] and Lenco and Seblewongel [21]. Moreover, cleaning frequency of twice (90 %), once (8 %) and thrice (2 %) a day was reported [21].

**Table 10:** Role of milk, milking hygiene and sanitation in study area.

Variables	Chena (192)	Gesha (192)	Total average	Chi-square	P value
Aesthetic	17.70 %	16.70 %	17.20 %	4.02	0.40(NS)
Home consumption	36.50 %	35.40 %	35.90 %		
Medication	16.10 %	20.30 %	18.20 %		
Economic source	29.70 %	27.60 %	28.60 %		
**Cleaning frequency**					
Once	68.20 %	69.30 %	68.80 %	1.215	0.545(NS)
Twice	20.30 %	16.70 %	18.50 %		
Conditional	11.50 %	14.10 %	12.80 %		

Significantly different at * p˂0.05 and **p˂0.01.

#### 3.4.9 Milking round, impacts of parity and lactation stages

Milking was once (22.7 %), twice (70.6 %), and three times per day (6.8 %) with highly significant variation between districts (Table 11). There were also highly significant (p˂0.01) differences between milking rounds and lactation stages recorded for the current study. In agreement with the present finding, Yitaye et al. [67] reported that a higher proportion of farmers (83.8 %) milked their cows twice a day. Contrarily, Once and thrice per day milking frequency was reported [68]. Lenco and Seblewongel [21] reported milking frequency of twice (90 %), once (8 %) and thrice (2 %) a day.

**Table 11:** Milking round and impacts lactation stages.

Milking Round	District				Lactation stage				
	Gesha	Chena	Chi	P value	Early	Mid	Late	Chi	P value
Once	15.10 %	30.20 %	16.27	0.00 (**)	18.30 %	0.00 %	50.80 %	154.20	00.00 (**)
Twice	75.00 %	66.10 %			61.10 %	100.00 %	49.20 %		
Three	9.90 %	3.60 %			20.60 %	0.00 %	0.00 %		

Significantly different at **p˂0.01.

### 3.5 Opportunities of dairy production in study area

Opportunities for indigenous dairy production in the study area were presented in Table 12. As a friendly result, Tadesse and Mengistie [69] reported that, income generation, research institution establishment, employment, land inputs and traction, milk consumption, livestock genetic resource abundance and indigenous knowledge improvement were prioritized opportunities. Moreover, cattle market expansion, government considerations and supports, infrastructure availability, and veterinary service provisions were declared as opportunities [70]. Similarly, genetic resources and biodiversity endowers, establishment of several structures and service centers such as veterinary health and artificial insemination (AI) centers, extensive service of agricultural extension, income growth and availability of trained manpower, research institutions, and technologies were reported as opportunities behind dairy cow production [71]. Comparably, economic development, food sources and employment opportunities were reported [72]. Research and academic opportunities, food, ventures, technological advancements, and genetic resource diversities were opportunities synergetic with the current study result [73]. Furthermore, veterinary service expansion, marketing accessibility, and soil fertility were favored opportunities ranked in descending order [19].

**Table 12:** Opportunities of indigenous dairy cow production.

Opportunities	Gesha (192)		Chena (192)	
	Index	Rank	Index	Rank
Agricultural machinery and land inputs access	0.25	1st	0.229	1st
Provision of balanced food	0.214	2nd	0.191	2nd
Income generation and Employment fortunes	0.178	3rd	0.171	3rd
Prestige and social respect	0.113	4th	0.09	5th
Research Institution and wisdom expansion	0.059	6th	0.061	6th
Genetic bio diversification	0.087	5th	0.049	7th
Biotechnology and factory advancement	0.043	8th	0.02	8th
Emblem of Fame	0.056	7th	0.111	4th

## 4 Conclusions

Dairy production system of the study area was a mixed crop-livestock production system in which uncharacterized zebu cows were burdened with production responsibilities. Trait preferences which rely on indigenous knowledge of producers, were in favor of the selection criteria. Production performances were below international production performance standards, eventhough they were better than the national average yield. The study also showed that the over all daily milk yield, lactation milk yield, and lactation length were highly influenced by district, parity, seasons and their interactions. Even though indigenous dairy cows were powerhouses of opportunities, welfare abandonment, poor management besides of constraints made them deprived in production aptitudes under ideal standards. Hence, rural dairy cow producers should have to get awareness creation training, and periodic proficiency curriculum for veterinarians and technicians needs to be established. Constraints demanding immediate alleviation should get priority, then identified, and an unconditional reaction has to be given.

## Ethical approval and consent to participate

Animal care and ethical issue were careful evaluated and approved the experiment (1956 ET-12/2020) by MizanTepi University, College of Agriculture and Natural Resources ethics Committee. Directive 2010/63/EU of the European Union guidelines (2010) concerning the treatment and use of animals in research and development purposes were employed.

## Availability of data and materials

The data that has been used is confidential.

## Funding

No funding was obtained for this study.

## Additional information

No additional information is available for this paper.

## CRediT authorship contribution statement

**Regasa Begna Roba:** Conceptualization, Data curation, Formal analysis, Investigation, Methodology, Project administration, Software, Supervision, Validation, Visualization, Writing – original draft, Writing – review & editing. **Yakob Asfaw:** Conceptualization, Data curation, Formal analysis, Investigation, Methodology, Resources, Supervision, Validation, Visualization, Writing – original draft, Writing – review & editing. **Worku Masho Bedane:** Conceptualization, Data curation, Methodology, Supervision, Visualization, Writing – original draft, Writing – review & editing.

## Declaration of competing interest

The authors declare that they have no known competing financial interests or personal relationships that could have appeared to influence the work reported in this paper.
